# Artesunate induces mitochondria-mediated apoptosis of human retinoblastoma cells by upregulating Kruppel-like factor 6

**DOI:** 10.1038/s41419-019-2084-1

**Published:** 2019-11-13

**Authors:** Ying Yang, Nandan Wu, Yihui Wu, Haoting Chen, Jin Qiu, Xiaobing Qian, Jieting Zeng, Kin Chiu, Qianying Gao, Jing Zhuang

**Affiliations:** 1 State Key Laboratory of Ophthalmology, Zhongshan Ophthalmic Center, Sun Yat-sen University, Guangzhou, 510060 P. R. China; 2 Department of Ophthalmology, The University of Hong Kong, Hong Kong SAR, P. R. China; 3 Department of Ophthalmology, The 2nd Affiliate Hospital, Wenzhou Medical University, Wenzhou, 325000 P. R. China

**Keywords:** Eye cancer, Drug delivery

## Abstract

Retinoblastoma (RB) is the most common primary intraocular malignancy in children. Intravitreal chemotherapy achieves favorable clinical outcomes in controlling RB vitreous seeds, which are a common reason for treatment failure. Thus, a novel, effective and safe intravitreal chemotherapeutic drug is urgently required. The malaria drug artesunate (ART) recently demonstrated remarkable anticancer effects with mild side effects. The purpose of this study is to investigate the anti-RB efficacy, the underlying mechanism and the intraocular safety of ART. Herein, we verified that ART inhibits RB cell viability and induces cell apoptosis in a dose- and time-dependent manner. Microarray analysis revealed that Kruppel-like factor 6 (KLF6) was upregulated after ART treatment, and this was further confirmed by real-time PCR and western blot assays. Silencing of KLF6 expression significantly reversed ART-induced RB cell growth inhibition and apoptosis. Furthermore, ART activated mitochondria-mediated apoptosis of RB cells, while silencing KLF6 expression significantly inhibited this effect. In murine xenotransplantation models of RB, we further confirmed that ART inhibits RB tumor growth, induces tumor cell apoptosis and upregulates KLF6 expression. In addition, KLF6 silencing attenuates ART-mediated inhibition of tumor growth in vivo. Furthermore, we proved that intravitreal injection of ART in Sprague-Dawley (SD) rats is safe, with no obvious retinal function damage or structural disorders observed by electrophysiology (ERG), fundal photographs, fundus fluorescein angiography (FFA) or optical coherence tomography (OCT) examinations. Collectively, our study revealed that ART induces mitochondrial apoptosis of RB cells via upregulating KLF6, and our results may extend the application of ART to the clinic as an effective and safe intravitreal chemotherapeutic drug to treat RB, especially RB with vitreous seeds.

## Introduction

Retinoblastoma (RB) is the most common childhood cancer of the eye; it arises from the retina and causes serious damage to vision, even endangering lives^1^. The current standard treatment for RB includes thermotherapy, cryotherapy, radiotherapy, surgery, and chemotherapy^2^. RB is a chemosensitive tumor. Although chemotherapy is widely used in the clinic and achieves a good therapeutic effect, vitreous seeds are still a main reason for treatment failure^3,4^. Many recent studies have suggested that intravitreal chemotherapy achieves good control of RB vitreous seeds and no serious systemic side effect was observed^5–7^. Intravitreal injection of carboplatin, melphalan, and topotecan results in outstanding vitreous seed control, but ocular complications, including retinal pigment epithelial alterations, retinal vasculitis, transient vitreous hemorrhage and paraxial posterior lens opacity, cannot be ignored^8–12^. Thus, a new, effective and safe intravitreal chemotherapeutic drug is urgently required for the treatment of RB, especially with vitreous seeds.

Artemisinin is a compound extracted from the Chinese herb qinghao and has been widely used in the clinic to treat malaria^13^. Artesunate (ART), a semisynthetic derivative of artemisinin, has the advantages of long half-life, good water solubility and low toxicity compared with artemisinin^14^. Currently, accumulating evidence has demonstrated that ART effectively inhibits the growth of various cancer cells, including leukemia, renal cell carcinoma, esophageal cancer, ovarian cancer, and RB^15–19^. In addition to cell and animal experiments, the antitumor effect and safety of ART has already been verified in patients. Zhang et al.^20^ reported that the combination of ART with vinorelbine and cisplatin can elevate outcomes in advanced non-small-cell lung cancer patients without additional side effects. Many studies have explored the possible antitumor mechanisms of ART, such as cell cycle arrest, induction of cell apoptosis, regulation of tumor-related gene expression, and inhibition of angiogensis^21,22^. However, the underlying molecular mechanism of ART action on RB cells remains unclear.

Moreover, an ideal intravitreal chemotherapeutic drug for the treatment of RB should possess excellent antitumor effects and provide a favorable safety profile. Therefore, the aims of the present study are (1) to investigate the anti-RB efficacy and the underlying antitumor mechanism of ART in vitro and in vivo; and (2) to explore the ocular safety of intravitreal injection of ART. Our results reveal a new molecular antitumor mechanism of ART on RB, and provide evidence to verify that ART may serve as an effective and safe intravitreal chemotherapeutic drug to treat intraocular RB, especially RB with vitreous seeds.

## Results

### ART inhibits cell proliferation and induces apoptosis in RB cells

**Fig. 1:**
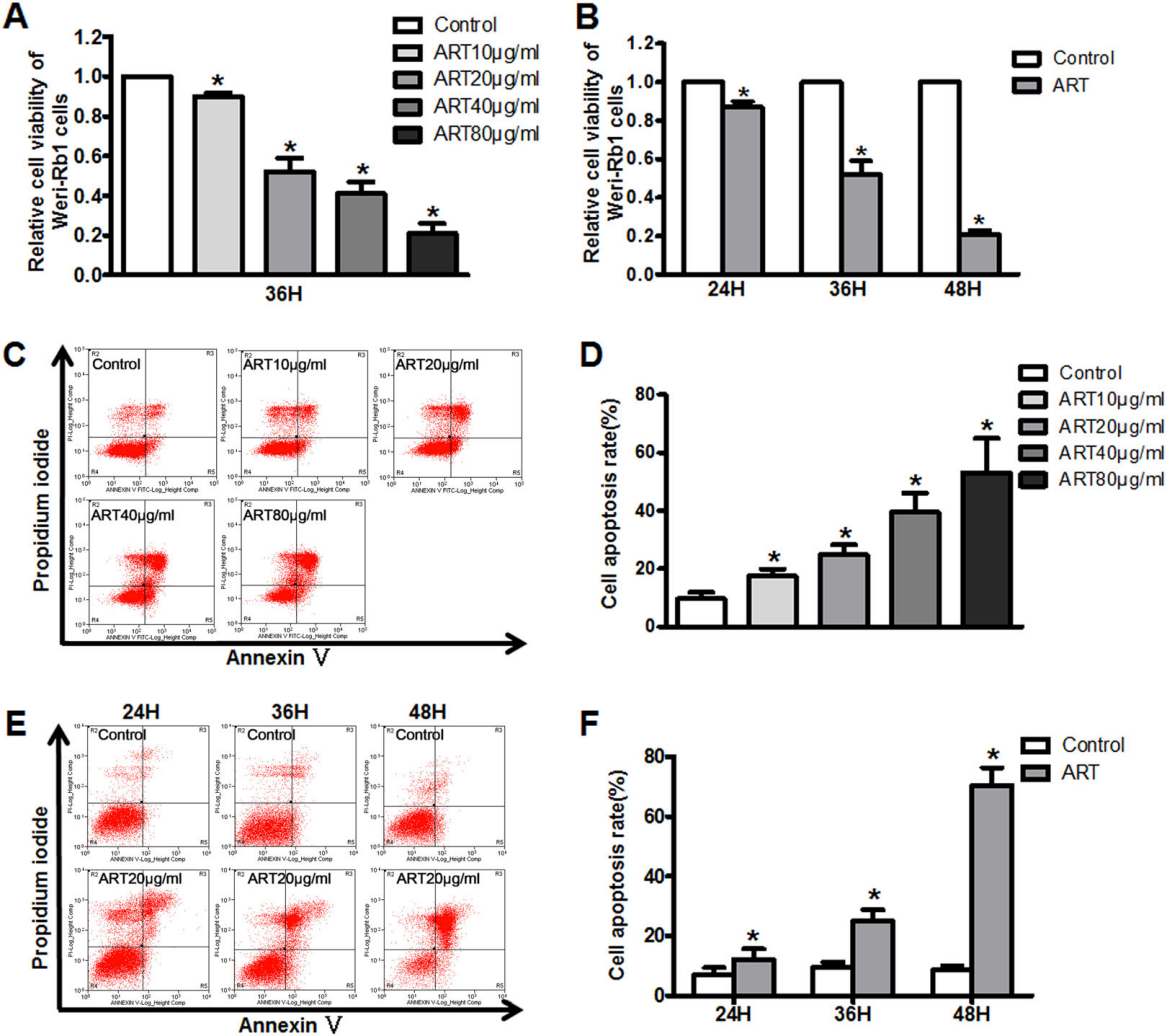
ART inhibits WERI-Rb1 cells proliferation and induces cell apoptosis. **a** The growth of WERI-Rb1 cells was inhibited by ART treatment in a dose-dependent manner. **b** ART also exerted a time-dependent growth inhibition on WERI-Rb1 cells. **c**, **d** ART treatment promoted cell apoptosis in a dose-dependent manner, the percentages of apoptotic WERI-Rb1 cells were presented as histograms. **e**, **f** The percentages of apoptotic WERI-Rb1 cells were also increased follow the treated time extending. All results are presented as the mean ± SD (*n* = 3, *P < 0.05, vs. control)

Previous studies have suggested that ART inhibits the proliferation of many types of cancer cells^21,22^. Our result showed that ART inhibited WERI-Rb1 cell proliferation in a dose-dependent manner, the cell viability rates were as follows: Control: 1, ART: 0.9 ± 0.02 (10 μg/ml), 0.52 ± 0.07 (20 μg/ml), 0.41 ± 0.06 (40 μg/ml) and 0.21 ± 0.05 (80 μg/ml) (Fig. 1a). ART also exerted time-dependent growth inhibition, and the cell viability rates were as follows: Control: 1, 0.87 ± 0.03 (24 h), 0.52 ± 0.07 (36 h), 0.21 ± 0.02 (48 h) (Fig. 1b). In addition, Y-79 cells also exhibited a dose- and time-dependent response to ART (Supplementary Fig. S1). However, ART had a limited inhibitory effect in normal retina cells, such as the A-RPE 19 cells and primary rat retina neurons (Supplementary Fig. S2). Thus, ART may be a promising intravitreal chemotherapeutic drug to treat RB.

To confirm whether ART-induced cell growth inhibition is associated with apoptosis, flow cytometry analysis was performed. Figure 1c, d shows that ART significantly promotes WERI-Rb1 cell apoptosis in a dose-dependent manner, the percentages of apoptotic cells as follows: Control: 9.7 ± 1.5%, ART: 17.8 ± 3.3% (10 μg/ml), 24.9 ± 3.8% (20 μg/ml), 39.6 ± 9.2% (40 μg/ml), and 53.2 ± 3.3% (80 μg/ml). Subsequently, a time-response experiment was performed, and with the extended exposure time to ART, the rate of apoptosis increased, the cell apoptosis rate as follows: 24 h, Control: 7.2 ± 2.2%, ART: 12.2 ± 3.4%; 36 h, Control: 9.7 ± 1.5%, ART: 24.9 ± 3.8%; 48 h, Control: 8.6 ± 1.5%, ART: 70.6 ± 5.8% (Fig. 1e, f).

### ART induces apoptosis of RB cells by upregulating KLF6

**Fig. 2:**
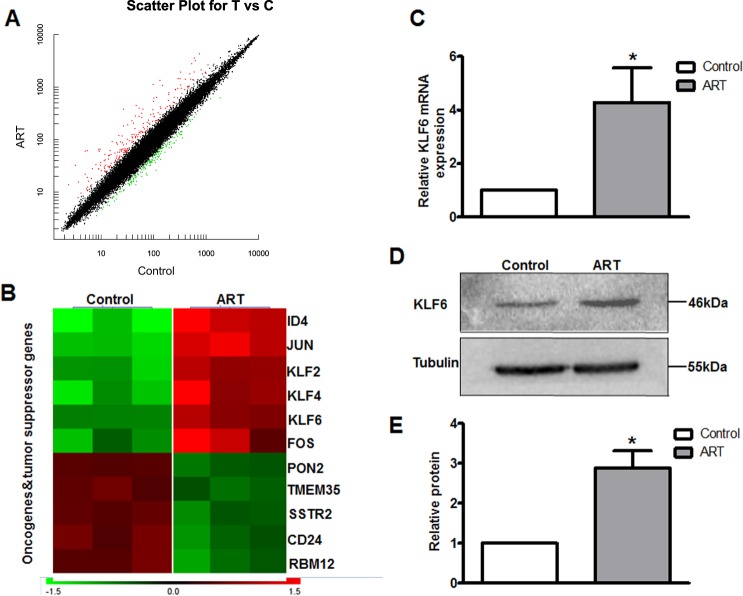
ART treatment increases the KLF6 expression in WERI-Rb1 cells. **a** Microarray analysis was used to screen altered genes in WERI-Rb1 cells after ART treatment. Black dots, red dots and green dots referred to stably, upregulated, and downregulated genes, respectively. **b** Several cancer oncogenes and suppressors were selected to further confirmation. Through real-time PCR and western blot assay, a tumor suppressor KLF6, both mRNA (**c**) and protein (**d**, **e**) expression were upregulated after ART treatment. All results are presented as the mean ± SD (*n* = 3, *P < 0.05, vs. control)

To understand the molecular mechanism of ART-induced apoptosis in RB cells, we compared the gene expression profiles between control and ART-treated WERI-Rb1 cells using gene microarray analysis (Fig. 2a). Among these altered genes, some oncogenes, such as PON2 and CD34, were downregulated, and some tumor suppressors, such as KLF6 and JUN were upregulated (Fig. 2b). Using real-time PCR and western blot assays for further verification, we found that KLF6, which is known as a tumor suppressor that induces apoptosis in various cancer cells^23,24^, was upregulated in both RNA and protein expression after ART treatment (Fig. 2c–e). Therefore, KLF6 may be involved in ART-induced RB cell apoptosis.

**Fig. 3:**
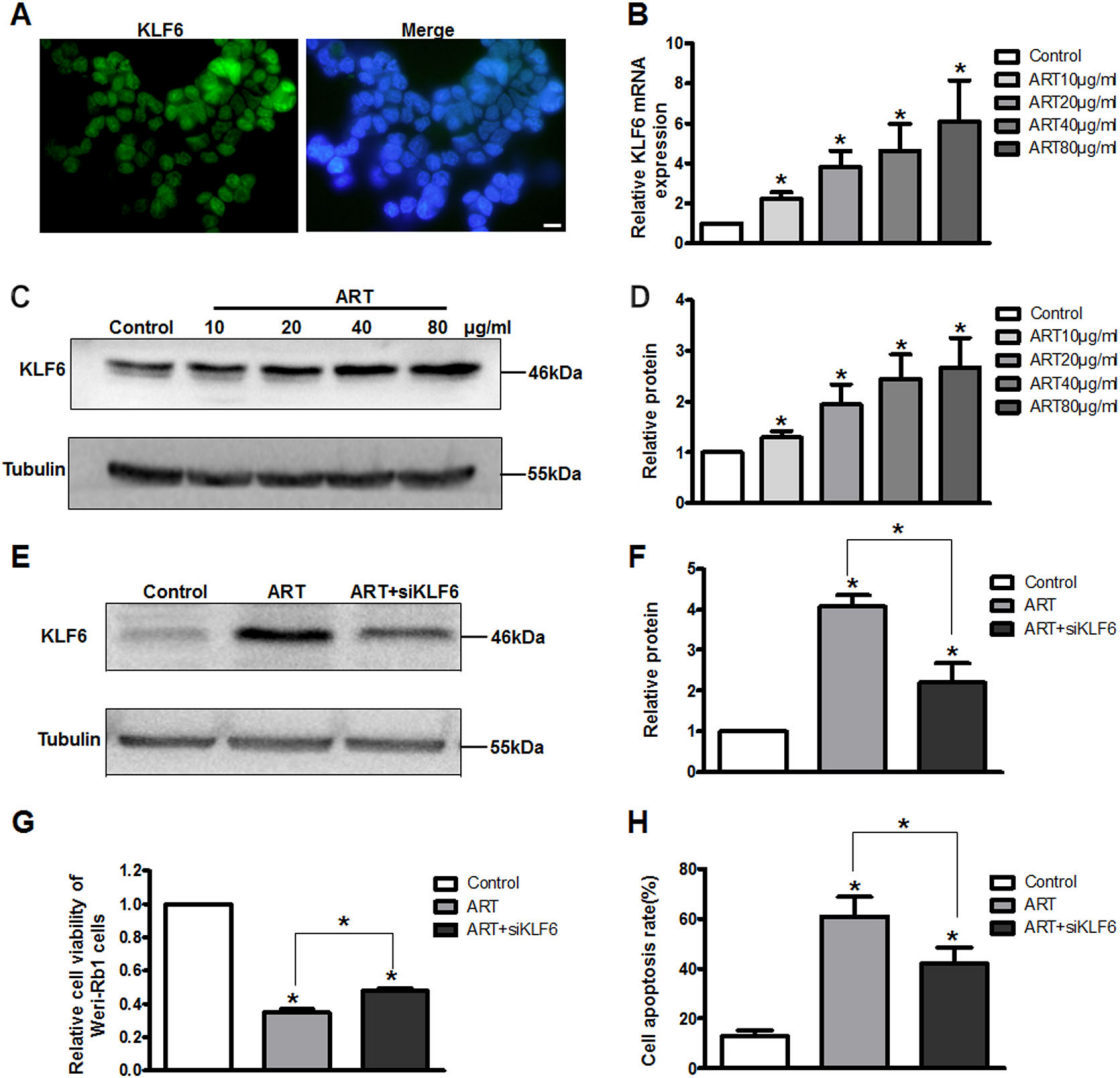
ART-induced apoptosis of WERI-Rb1 cells by upregulating KLF6. **a** Endogenous location of KLF6 (green) in WERI-Rb1 cells was detected by immunohistofluorescence. Original magnification, ×100; scale bar, 10 μm. Real-time PCR (**b**) and western blot analysis (**c**, **d**) showed that KLF6 mRNA and protein expression were markedly upregulated after ART treatment in a dose-dependent manner. **e**, **f** Western blot analysis showed that KLF6 was decreased after transfection with siRNA-KLF6. **g**, **h** Silenced KLF6 expression could significantly reverse ART-induced cell growth inhibition and apoptosis. All results are presented as the mean ± SD (*n* = 3, *P < 0.05 vs. control)

Subsequently, KLF6 was detected mainly in the nuclei of WERI-Rb1 cells by immunohistofluorescence (Fig. 3a). We also measured KLF6 mRNA and protein levels in WERI-Rb1 cells after the addition of different concentrations of ART for 24 h. The mRNA expression of KLF6 in WERI-Rb1 cells increased in a dose-dependent manner (by 2.22-, 3.82-, 4.61-, and 6.08-fold, respectively, compared with the control) (Fig. 3b). Similarly, altered KLF6 protein expression was consistent with the change in mRNA level (by 1.3-, 1.94-, 2.45-, and 2.67-fold, respectively, compared with the control) (Fig. 3c, d).

To further confirm that KLF6 plays a crucial role in ART-induced WERI-Rb1 cell apoptosis, siRNA transfection was performed to silence KLF6 expression after ART treatment. As shown in Fig. 3e, f, this markedly decreased KLF6 expression (Control: 1; ART: 4.07 ± 0.3; ART + siKLF6: 2.2 ± 0.47; *P* < 0.05). Furthermore, silencing of KLF6 significantly increased cell viability (Control: 1; ART: 0.35 ± 0.02; ART + siKLF6: 0.48 ± 0.015; *P* < 0.05) and attenuated cell apoptosis (Control: 12.77 ± 2.53%; ART: 60.85 ± 7.97%; ART + siKLF6: 41.96 ± 6.51; *P* < 0.05) following ART treatment in WERI-Rb1 cells (Fig. 3g, h).

Similar results were also confirmed in Y-79 cells (Supplementary Fig. S4A–G). These results indicate that ART induces apoptosis of RB cells via upregulating KLF6.

### KLF6 is involved in ART-induced mitochondrial apoptosis of RB cells

**Fig. 4:**
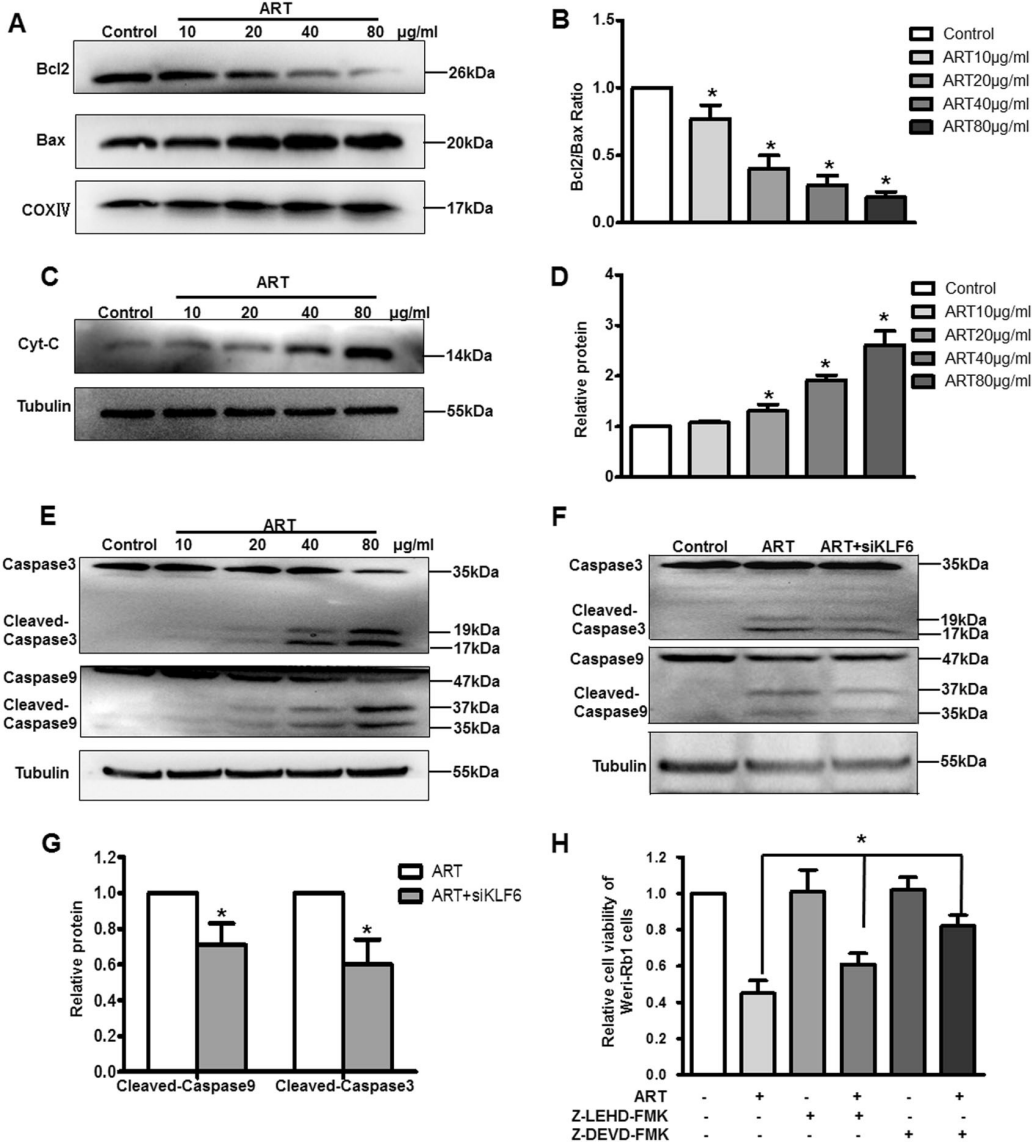
KLF6 is involved in ART-induced apoptosis of WERI-Rb1 cells via the mitochondrial signaling pathway. **a**, **b** Mitochondrial Bax and Bcl-2 expression were detected and quantified, the Bcl2/Bax ratio was decreased after ART treatment. COXIV was used as a loading control. **c**, **d** Cytosolic cytochrome C expression was increased after ART treatment. β-Tubulin was used as a loading control. **e** ART promoted cleavage of caspases-9 and -3. β-Tubulin was used as a loading control. **f**, **g** Silencing of KLF6 significantly inhibited cleavage of caspases-9 and -3 detected by western blot and quantified. **h** Two caspase-specific inhibitors Z-LEHD-FMK and Z-DEVD-FMK reversed ART-induced apoptosis. All results are presented as the mean ± SD (*n* = 3, *P < 0.05, vs. control)

Cell apoptosis is mainly triggered through the extrinsic or the intrinsic pathway^25^. It was reported that KLF6 is associated with mitochondrial function in the kidney^26^, so several key mitochondrial apoptosis-related genes were examined by western blot analysis. ART downregulated mitochondrial Bcl-2 expression, upregulated Bax expression, and decreased the Bcl-2/Bax ratio (Control: 1, ART: 0.77 ± 0.1 (10 μg/ml), 0.4 ± 0.1 (20 μg/ml), 0.28 ± 0.07 (40 μg/ml), and 0.19 ± 0.04 (80 μg/ml)) (Fig. 4a, b). ART also promoted the release of cytochrome c (cyt-c) into the cytosol (Control: 1; ART: 1.08 ± 0.03 (10 μg/ml), 1.31 ± 0.13 (20 μg/ml), 1.92 ± 0.1 (40 μg/ml), and 2.61 ± 0.28 (80 μg/ml)) (Fig. 4c, d). Subsequently, cytosol cyt-c stimulated the cleavage of caspases-9 and -3 (Fig. 4e), suggesting that ART induces cell apoptosis partly by activating the mitochondria-mediated pathway.

Caspases-9 and -3 are the most important downstream effector caspases in mitochondria-mediated apoptosis, so we detected their expression after silencing KLF6 followed by ART treatment in WERI-Rb1 and Y-79 cells. The results showed that the expression of cleaved caspase-3 and cleaved caspase-9 were increased after ART treatment; however, silencing of KLF6 significantly inhibited this effect (Fig. 4f, g) (Supplementary Fig. S4H-I). Moreover, both caspase-9 (Z-LEHD-FMK) and caspase-3 (Z-DEVD-FMK)-specific inhibitors could reverse ART-induced apoptosis (relative cell viability value: Control: 1; ART: 0.45 ± 0.07; ART + Z-LEHD-FMK: 0.61 ± 0.06; and ART + Z-DEVD-FMK: 0.82 ± 0.06; *P* < 0.05) (Fig. 4h). Taken together, these data indicate that KLF6 plays a crucial role in ART-induced mitochondrial apoptosis in RB cells.

### ART induces RB tumor cells apoptosis and upregulates KLF6 in vivo

Based on the in vitro results, the RB orthotopic xenotransplantation model and subcutaneous xenotransplantation model were established to determine the anti-RB efficacy and the molecular mechanism of ART.

**Fig. 5:**
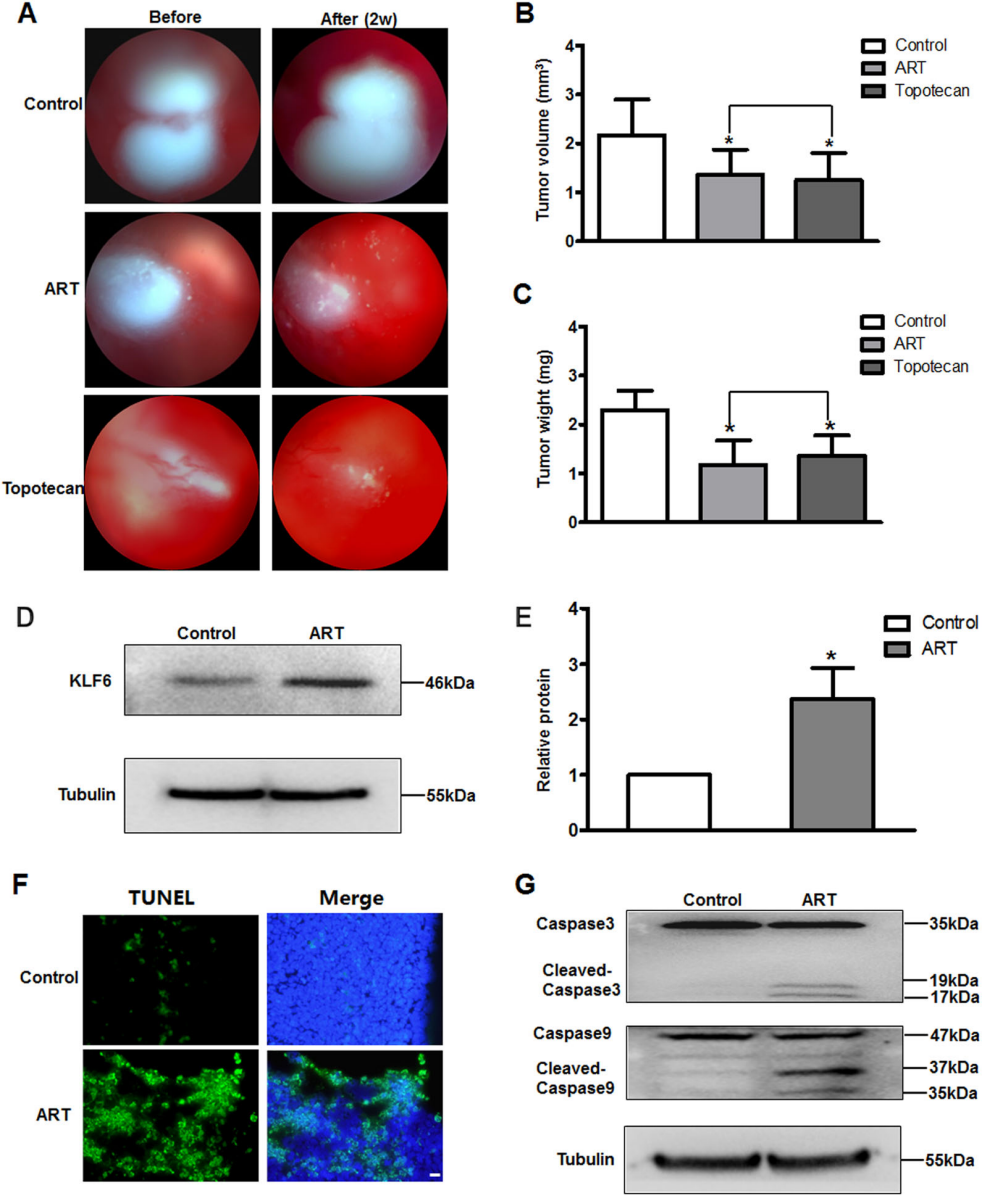
ART induces RB tumor cells apoptosis and upregulates KLF6 in vivo. **a** Fundus photography images before and 2 weeks after injection. Tumor volume (**b**) and tumor weight (**c**) were significantly higher in the control group compared to the ART and topotecan treatment groups (*n* = 6, *P < 0.05 vs. control). **d** Extraction of tumor tissue protein to detect expression changes of KLF6 between saline- and ART-injected mice. **e** Relative quantification was performed by densitometry. **f** Fluorescence photomicrographs of TUNEL staining (green) in tumor tissue sections of the RB model. Original magnification, ×40; scale bar, 200 μm. **g** Extraction of tumor tissue protein to detect protein expression changes of cleaved caspase-9 and caspase-3 between saline- and ART-injected mice

On RB orthotopic xenotransplanted mice models, after intravitreal injection for two weeks, the tumor growth was significantly inhibited in ART- and topotecan-treated mice, but tumor growth increased in saline-treated mice compared with tumor size before injection according to color fundus photography (Fig. 5a). The eyes were then extracted, and tumor volume and weight were measured. Tumor volume was significantly less in the ART-treated (1.25 ± 0.56 mm^3^) and topotecan-treated (1.36 ± 0.52 mm^3^) groups relative to saline-treated mice (2.17 ± 0.73 mm^3^) (Fig. 5b). Tumor weight was dramatically reduced in the ART-treated (1.18 ± 0.5 mg) and topotecan-treated (1.36 ± 0.52 mg) groups compared with the saline-treated group (2.30 ± 0.39 mg) (Fig. 5c). Moreover, there was no difference between tumor volume and tumor weight between the ART- and topotecan-injected mice (*P* > 0.05), suggesting that ART inhibits tumor growth as effectively as topotecan.

Furthermore, KLF6 protein expression was significantly increased in the tumors of ART-treated mice compared with control mice (Control: 1; ART: 2.37 ± 0.56; *P* < 0.05) (Fig. 5d, e). We also evaluated the effects of ART on the induction of apoptosis and apoptosis-related genes in tumor xenografts. The number of apoptotic (TUNEL-positive) cells was significantly increased in the tumors of ART-treated mice compared with saline-treated mice (Fig. 5f). Western blot analysis showed that ART treatment resulted in the activation of caspases-9 and -3 in vivo (Fig. 5g).

**Fig. 6:**
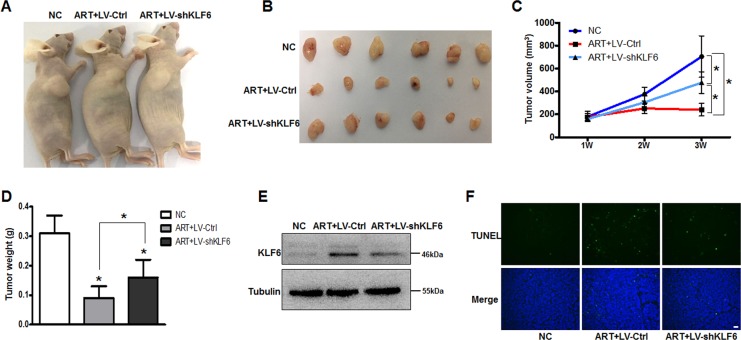
KLF6 silencing attenuates the effect of ART on inhibiting tumor growth in vivo. **a**, **b** Macroscopic appearance of tumors after intraperitoneal injection saline or ART for 2 weeks. **c** The tumor growth curve in the xenotransplantation model (*n* = 6). **d** Tumor weight of mice from each group was measured after intraperitoneal injection for two weeks (*n* = 6). **e** Western blot assay was performed to detect expression changes of KLF6 among three groups. **f** Fluorescence photomicrographs of TUNEL staining (green) in tumor tissue sections of the RB model. Original magnification, ×40; scale bar, 200 μm. *P < 0.05 vs. control. NC negative control

To further verify that KLF6 plays a crucial role in ART-induced RB tumor growth, we constructed subcutaneous RB models and intraperitoneal injected with ART (50 mg/kg) or saline for 2 weeks. Macroscopically, the tumors from ART-treated mice (groups 2(ART + LV-Ctrl) and 3(ART + LV-shKLF6)) appeared smaller than those from the saline-treated mice (group 1(NC)). However, the tumors from group 3 were slightly larger than the tumors from group 2 (Fig. 6a, b). As shown in Fig. 6c, tumor volumes from the 3 groups showed no difference 1 week after WERI-Rb1 cell injection (NC: 181 ± 45 mm^3^, ART + LV-Ctrl: 176 ± 31 mm^3^, ART + LV-shKLF6: 160 ± 23 mm^3^). After intraperitoneal injection for 2 weeks, the tumor volumes of group 2 remained unchanged from the original volumes, and the tumor volumes of group 3 were slightly larger than their starting volumes, whereas the tumor volumes of group 1 were much bigger than before (NC: 705 ± 180 mm^3^, ART + LV-Ctrl: 241 ± 54 mm^3^, ART + LV-shKLF6: 478 ± 94 mm^3^). The change in tumor weights also showed that ART treatment significantly inhibited tumor growth and that KLF6 silencing attenuated the effect of ART (NC: 0.31 ± 0.06 g, ART + LV-Ctrl: 0.09 ± 0.04 g, and ART + LV-shKLF6: 0.16 ± 0.06 g) (Fig. 6d). We also detected KLF6 protein expression among three groups, the results showed that ART treatment could increase KLF6 expression in vivo, but LV-shKLF6 infection inhibited the effect, suggesting that the KLF6 knockout model was successfully established (Fig. 6e). Furthermore, TUNEL assay showed that KLF6 silencing could attenuate the tumor cells apoptosis rate following ART treatment (Fig. 6f).

These results were consistent with our data from WERI-Rb1 cells in vitro, further confirming that ART administration induces mitochondria-mediated apoptosis in RB cells via upregulating KLF6.

### Safety evaluation of intravitreal injection of ART

**Fig. 7:**
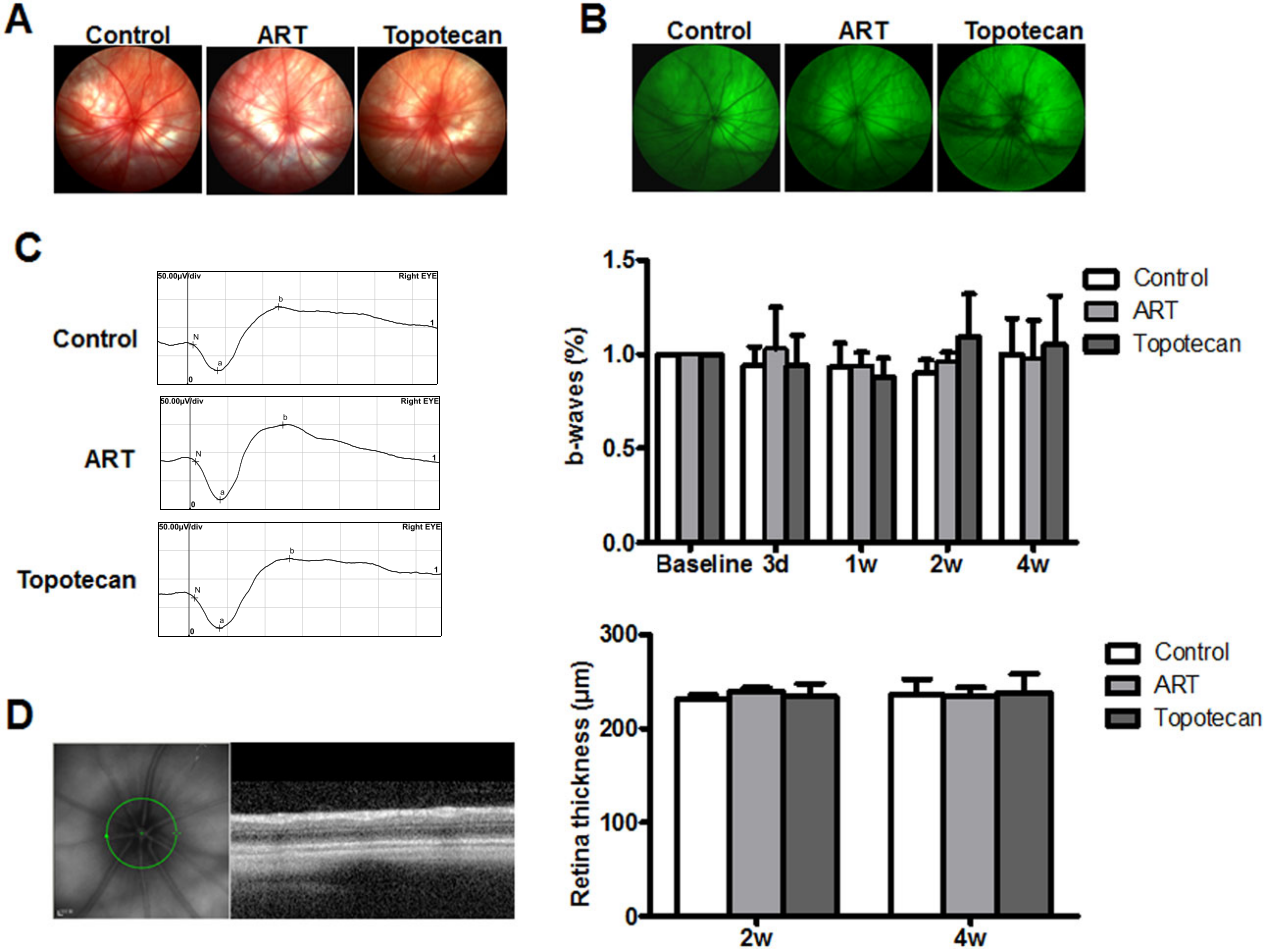
Safety evaluation of intravitreal injection of ART. **a**, **b** Fundus photography and FFA images of saline-, ART- and topotecan-injected rats. **c** ERG examination was performed, and b-waves were recorded after vitreous injection at 3 days, 1 week, 2 weeks and 4 weeks. **d** OCT examination was performed after vitreous injection at 2 weeks and 4 weeks, and the retina thickness was analyzed. All results are presented as the mean ± SD (*n* = 5, *P < 0.05, vs. control)

The safety of ART intravitreal injection was determined in SD rats, with topotecan treatment serving as a positive control. First, images of color fundus photography and fundus fluorescein angiography (FFA) did not reveal retinal vascular dilatation, tortuosity or fluorescein leakage after 4 weeks of injection of saline, ART or topotecan (Fig. 7a, b). We then compared the ERG of eyes before and after injection of saline, ART or topotecan at 3 days, 1 week, 2 weeks and 4 weeks, and found no significant changes in b-waves between the three groups (Fig. 7c) (*P* > 0.05). Moreover, OCT results also did not show obvious retinopathy, and the retinal thickness was not significantly different among saline-, ART- and topotecan-injected eyes (Fig. 7d). In conclusion, the results suggested that ART is safe and exhibits good efficacy profiles for the treatment of RB.

## Discussion

ART, a powerful and safe antimalarial drug, recently demonstrated remarkable anticancer effects^21,22^. Here, our results confirmed that ART has excellent anti-RB efficacy and has a favorable safety profile in vitro and in vivo. Furthermore, we revealed a novel anti-RB mechanism of ART: ART treatment upregulates KLF6 expression, which causes mitochondrial dysfunction, increases the Bax/Bcl-2 ratio, promotes the release of cyt-c, and stimulates the cleavage of caspase-9 and caspase-3, resulting in cell apoptosis (Supplementary Fig. S6).

In the present study, we found that ART treatment significantly inhibited RB cell viability in a dose- and time- dependent manner. Many events may result in cell growth inhibition, such as cell cycle arrest, apoptosis and necrosis. Our data showed that ART only induces arrest of the cell cycle at the S phase and necrosis in a small number of WERI-Rb1 cells; however, it dramatically causes cell apoptosis (Supplementary Fig. S3). In addition, ART also promoted cell apoptosis in a dose- and time-dependent manner. Therefore, ART suppresses RB cell growth mainly by inducing apoptosis.

Apoptosis is a form of programmed cell death that can be seen as an important barrier to developing cancer, and the activation of apoptosis is a crucial mechanism for most chemotherapy drugs^27^. It was reported that ART inhibits the growth of human MCF-7 breast cancer cells, SGC-7901 gastric cancer cells, and HCT116 colon cancer cells by inducing apoptosis^28–30^. In addition, we further found that ART induces RB cell apoptosis by upregulating KLF6.

KLF6, a zinc finger transcription factor, is a tumor suppressor and plays an important role in the development and metastasis of various human cancers. Recent studies have reported that KLF6 could serve as a new molecular marker candidate for tumor prognosis and a potential therapeutic target, because abnormal KLF6 expression was associated with a poor clinical prognosis, cancer recurrence, and drug resistance^31–34^. The overexpression of KLF6 induced apoptosis in non-small-cell lung cancer and prostate cancer cells^23,24^. In this study, ART treatment could significantly increase KLF6 expression, and silencing of KLF6 could reverse ART-induced cell growth inhibition and apoptosis in RB cells. Furthermore, overexpression of KLF6 via FLAG-hKLF6 plasmid transfection inhibited WERI-Rb1 cell growth and promoted cell apoptosis (Supplementary Fig S5). Together, the results suggest that KLF6 plays a critical role in the apoptosis of RB cells caused by ART treatment.

KLF6 was reported as a crucial mitochondrial regulator in the kidney^26^. Our data showed that ART increases the mitochondrial Bax/Bcl-2 ratio, promotes the release of cyt-c, and stimulates the cleavage of caspase-3 and caspase-9, demonstrating that the mitochondria-mediated pathway is involved in ART-induced WERI-Rb1 cell apoptosis. More importantly, silencing of KLF6 could inhibit the cleavage of caspase-3 and caspase-9, which is a critical process in the mitochondrial apoptosis pathway^35^. This further confirms that the overexpression of KLF6 leads to mitochondrial dysfunction and then activates mitochondria-mediated apoptosis in WERI-Rb1 cells after ART treatment. However, in cultured human podocytes, KLF6 knockdown resulted in activation of the intrinsic apoptotic pathway, which suggested that the function of KLF6 in normal cells is opposite to that in RB tumor cells. Additionally, a previous study suggested that the possible mechanism of KLF6-regulated cell apoptosis was by upregulating ATF3 expression in prostate cancer cells^23^, whereas the exact mechanism by which KLF6 regulates mitochondria-mediated apoptosis in WERI-Rb1 cells (whether directly caused by self-expression changes or indirectly via the regulation of downstream gene expression as a transcription factor) remains to be explored.

As a candidate intravitreal chemotherapeutic drug for RB treatment, we also confirmed that ART has excellent anti-RB efficacy with no obvious intraocular toxicity in vitro and in vivo. In this study, we used topotecan, a reported effective and safe intravitreal chemotherapeutic drug as a positive control for RB treatment^11^ and our results showed that ART (20 μg/ml) inhibits tumor cell growth as effectively as topotecan (6.7 μg/ml). For the safety assessment, we previously found that after intravitreal injection of ART in rabbit eyes, the drug concentrations detected in serum is far lower than the concentrations achieved for systemic antimalarial treatments (data not published). Furthermore, in the current study, the cytotoxic effect of ART on normal retinal cells was slight, and no obvious retina function damage or structural disorder was found by ERG, fundus photography, FFA, and OCT evaluation in SD rats after ART intravitreal injection. Therefore, intravitreal injection of ART demonstrates good systemic and intraocular safety.

In summary, this study elucidates a new underlying mechanism of the anti-RB mechanism of ART: ART induces mitochondria-mediated apoptosis of RB cells through upregulating KLF6. This study provides sufficient evidence to support ART as an ideal intravitreal drug for RB clinical treatment because it is highly effective, safe and inexpensive.

## Materials and methods

### Cell culture

The human RB cell lines WERI-Rb1, Y79 and the human retinal pigment epithelium cell line ARPE-19 were obtained from the American Type Culture Collection (ATCC, USA) and respectively cultured in RPMI-1640 and DMEM supplemented with 10% fetal bovine serum, 100 U/ml penicillin and 100 mg/ml streptomycin. For rat primary retina neurons culture, the protocol followed the method described by Yang^36^. All cells were cultured in a humidified atmosphere of 5% carbon dioxide at 37 °C. All cell culture medium/reagents were purchased from Gibco (Thermo Fisher, USA).

### Genes microarray

Microarray analysis was used Human Genome U133 Plus 2.0 Array (Affymetrix, CapitalBio Co., Ltd., Beijing, China) to screen changes in genome-wide gene expression patterns in WERI-RB1 cells between control group and ART treatment group. Three replicates were used for microarrays analysis.

### Cell viability assay

Cell treatment: ① Cultured WERI-Rb1 cells, Y79 cells, A-RPE 19 cells or primary rat retina neurons were exposed to various concentrations of ART (Guilin Pharmaceutical Co., Ltd., Guangxi, China) or a vehicle control at the indicated times. ② For RNA interference studies, The KLF6 siRNA and control sequences were as follows: KLF6-1: 5′-GCCTAGAGCTGGAACGTTA-3′; KLF6-2: 5′- GAAGATCTGTGGACCAAAA-3′; KLF6-3: 5′-GCCCGAGCTTTTGTTACAA-3′. The oligos were purchased from Ribobio (Guangzhou, China). The cells was pretreated with ART (20 μg/ml) for 12 h, then transfected with siRNAs using Lipofectamine RNAiMAX (Invitrogen, USA) according to the manufacturer’s protocol and allowed to incubate for 36 h. ③ For caspase inhibitor studies, WERI-Rb1 cells were pretreated with the 20 μM caspase-3 inhibitor Z-DEVD-FMK (Calbiochem, USA) or the caspase-9 inhibitor Z-LEHD-FMK (Calbiochem, USA) for 2 h, followed by treatment with ART (20 μg/ml) for 36 h. Finally, CCK-8 (Dojindo, Japan) reagent was added according to the manufacturer’s protocol. Subsequently, absorbance (optical density) was measured at 450 nm using a fluorescence plate reader (Power Wave XS) (BIO-TEK). Relative cell viability was determined by the optical density ratio of treated cells over the control.

### Flow cytometry

Cell treatment: ① Cultured WERI-Rb1 cells were exposed to various concentrations of ART or a vehicle control at the indicated times. ② Cultured WERI-Rb1 and Y-79 cells were pretreated with ART (20 μg/ml) treatment for 12 h, then transfected with KLF6-siRNA or control siRNA for 36 h. Apoptotic cells were double stained with fluorescein isothiocyanate (FITC)-annexin V and propidium iodide (PI) by using a FITC-Annexin V Apoptosis Detection Kit (BD Biosciences, USA) according to the manufacturer’s protocol and analyzed on a BD FACSort™ flow cytometer (BD Biosciences, USA)

### Real-time PCR assay

Treated cells was collected, total RNA was extracted using Trizol reagent (Invitrogen, USA) and 1 μl of total RNA was reverse transcribed for cDNA synthesis using a SYBR PrimeScript™ RT-PCR Kit (Takara, Dalian, China) according to the manufacturer’s protocol. The mRNA expression of KLF6 was detected by real-time PCR using a LightCycler480 II Sequence Detection System (Roche, Switzerland). Relative target gene expression was calculated using the ΔΔCt method^37^.

### Western blot assay

Treated cells and tissues were collected, and whole proteins were extracted by using RIPA total protein lysate kit (Biotech, Beijing, China). Mitochondrial and cytosolic fractions were isolated using the Mitochondria/Cytosol Fractionation Kit (Abcam, USA) following the manufacturer’s manual. Protein concentration was determined using the BCA Protein Assay Kit (Pierce, USA). Western blotting was carried out by standard protocols. The following primary antibodies were used: anti-tubulin (cat. no. 2148), anti-Bcl-2 (cat. no. 4223), anti-Bcl-2-associated X protein (Bax; cat. no. 5023), anti-caspase 9 (cat. no. 9502), and anti-caspase 3 (cat. no. 9665), all antibodies were purchased from Cell Signaling Technology, Inc. (Danvers, MA, USA). Anti-cytochrome C (cat. no. 66264-1-Ig) and anti-KLF6 (cat. no. 14716-1-AP) were obtained from Proteintech (Chicago, USA). Proteins were incubated with horseradish peroxidase (HRP)-conjugated anti-rabbit or anti-mouse IgG (CST, USA) and visualized with an enhanced chemiluminescence system. ImageJ densitometry software (National Institutes of Health, USA) was used for quantification.

### Immunohistofluorescence analysis of KLF6

Cultured WERI-Rb1 cells were fixed with ice-cold 4% PFA for 15 min and then blocked with 10% normal goat serum for 30 min. Sections were then incubated with a primary antibody against KLF6 overnight at 4 °C. Alexa Fluor 488 anti-rabbit IgG was used as a secondary antibody and nuclei were stained with DAPI.

### Animal studies

The animals used in this study were obtained from the Center of Experimental Animals of Sun Yat-sen University. The animal experimental procedures were performed in accordance with the ARVO Statement for the Use of Animals in Ophthalmic and Vision Research and were approved and monitored by the Institutional Animal Care and Use Committee of Zhongshan Ophthalmic Center (Permit Number: SYXK (YUE) 2017-076, 2017-093, 2019-009).

### A murine orthotopic xenotransplantation model of RB

Swiss background nu/nu mice (16–20 g) were used to establish an orthotopic xenotransplantation model of RB. 2 × 10^5^ WERI-Rb1 cells (1 μl) were injected into the vitreous of the right eyes of mice by using a Hamilton needle. The left eyes served as untreated controls. Approximately two weeks later, mice that achieved successful transplantation were randomly divided into three groups (*n* = 6): mice received vitreous injections of 1 μl of ART, topotecan or saline. The final concentration was expected to be ~20 μg/ml of ART or 6.7 μg/ml of topotecan. Topotecan^11^ served as the positive control. Before injection and two weeks after injection, all animals underwent color fundus photography (TRC-50DX; Topcon Co.) Subsequently, the globes were enucleated to evaluate tumor volume and tumor weight: tumor volume = right eyeball (volume)- left eyeball (volume); tumor weight = right eyeball (weight)- left eyeball (weight). Finally, eyeballs of the ART- and saline-injected groups were used for the terminal dUTP nick-end labeling (TUNEL) assay, or RB tissues were removed for protein extraction and western blot.

### Lentiviral infection and murine subcutaneous xenotransplantation model of RB

PLenti-KLF6-GFP-Puro and control recombinant lentiviruses were constructed by the Vigene Biosciences Company (Ji’nan, China). KLF6 siRNA sequences were as follows: siRNA-hKLF6-1: 5′-GCCTAGAGCTGGAACGTTA-3′; siRNA-hKLF6-2: 5′-GAAGATCTGTGGACCAAAA-3′. A total of 1 × 10^6^ WERI-Rb1 cells were seeded in a 6-well plate and cultured for 4 h and then infected with shKLF6 lentivirus (LV-shKLF6) or control lentivirus (LV-Ctrl) (multiplicity of infection (MOI) = 20). After infection for 12 h, the complex culture medium was replaced with virus-free complete medium.

Swiss background nu/nu mice (16–20 g) were used to establish a subcutaneous xenotransplantation model of RB. The animals were divided into three groups randomly (*n* = 6), mice in group 1 and 2 were injected with WERI-Rb1 cells that were infected with LV-Ctrl, and mice in group 3 were injected with WERI-Rb1 cells that were infected with LV- shKLF6. A total of 1.0 × 10^7^ infected WERI-Rb1 cells in 0.2 ml (Matrigel Matrix (BD Bioscience, San Jose, CA, USA) and RPMI-1640 with 20% FBS = 1:1) were injected subcutaneously into the right flanks of the mice. The tumor size was evaluated one week after injection. Then, the mice were injected intraperitoneally with ART (50 mg/kg) in groups 2 and 3 or an equal volume of saline in group 1 three times a week for 2 weeks. The tumor size was measured once weekly by caliper and calculated as tumor volume (the longest diameter x the shortest diameter^2^)/2. After intraperitoneally injection for 2 weeks, the tumors were harvested to be weighed and photographed. Then, the RB tissues were used for protein extraction and western blot. The degree of apoptosis in tumors was determined by using a TUNEL assay.

### TUNEL assay

Eyeballs and subcutaneous tumors of the xenotransplanted mouse model were embedded in optimal cutting temperature compound and cut into 7 μm sections. The degree of apoptosis was determined using a TUNEL kit (Roche, Switzerland) according to the manufacturer’s protocol. The concrete operation process followed the method described by Kim^38^. Sections were observed at ×40 magnification under a Nikon microscope (Nikon, Japan).

### Safety evaluation of intravitreal injection of ART

SD rats (180–220 g) were randomly divided into three groups (*n* = 5): mice received vitreous injections of ART (20 μg/ml), topotecan (6.7 μg/ml), or saline. Serial ophthalmologic examinations were performed before and after vitreous injection.

### Color fundus photography and FFA

Color fundus photography and FFA were performed on a fundus imaging system (MicronIV; PHOENIX, USA) at 4 weeks after vitreous injection. After animals were anesthetized and pupils were dilated, color fundus photography was performed. FFA images were recorded 5 min after intraperitoneal injection of fluorescein sodium (10%, 0.3 ml; Alcon).

### Electrophysiology (ERG)

To determine whether ART is harmful to the electrophysiological function of the retina, ERG was recorded before (baseline) and after vitreous injection at 3 days, 1 week, 2 weeks and 4 weeks. Standard procedures of ERG were followed our previous study^36^. The maximum dark-adapted b-waves amplitudes from each rat were recorded. To reduce individual variations in ERG amplitudes, a ratio of the amplitudes in the before and after injected eye of each rat were obtained for analysis.

### Optical coherence tomography (OCT)

To test whether the structure of the retina was altered by ART vitreous injection, total retinal thickness was measured by OCT (SpectralisOCT, Heidelberg, Germany) before and after vitreous injections at 2 and 4 weeks. After animals were anesthetized and pupils were dilated, the mean total retina was measured within a circle 3 mm in radius from the optical nerve head.

### Statistical analysis

All results are presented as the mean ± standard deviation. Student’s *t*-test (two groups) or analysis of variance (ANOVA, > two groups) was used to evaluate the significance of differences. *P* < 0.05 was considered statistically significant. SPSS 21.0 software was used for all statistical analyses.

## Supplementary information

Supplemental material

Supplementary Figure Legends

author contribution
