# Supplementary material for: Artesunate induces mitochondria-mediated apoptosis of human retinoblastoma cells by upregulating Kruppel-like factor 6
Source: Cell Death Dis. 2019 Nov 13;10(11):862. doi: 10.1038/s41419-019-2084-1 (PMC6853908; doi:10.1038/s41419-019-2084-1)
Supplement: Supplementary file 1 — Supplemental material [file 41419_2019_2084_MOESM1_ESM.docx]

**Supplementary material**

**Materials and methods**

**Cell cycle**

WERI-Rb1 cells (5×10^5^ cells/ml) were cultured into 6-well plates and treated with 40 μg/ml ART for 36h, then washed with ice-cold PBS and fixed with 75% ice-cold ethanol at 4°C overnight. Subsequently, the fixed cells were washed twice with PBS and incubated in PI staining solution for 30 min at 37°C. Fluorescent cells were analyzed on a flow cytometer.

**Plasmid transfection**

For KLF6 overexpression studies, WERI-Rb1 cells were transfected with 1.0 μg of vector pCI-neo or FLAG-KLF6 plasmids using Lipofectamine® 3000 Transfection Reagent (Invitrogen, Grand Island, NY, USA) according to the manufacturer’s protocol and allowed to incubate for 48 h. FLAG-KLF6 plasmids was a gift from Scott Friedman (Addgene, plasmid # 49489; http://n2t.net/addgene:49489; RRID:Addgene_49489). After cells treatment, transfection efficiency was detected by western blot, cell viability and cell apoptosis were evaluated by CCK-8 and flow cytometry assays, respectively.

**Results**

**ART inhibits retinoblastoma Y-79 cells viability in a dose- and time-dependent manner.**

To investigate the antiproliferative activity of ART on another human retinoblastoma cell line Y-79, cells were treated with various concentrations (10, 20, 40 or 80 μg/ml) of ART for 36 h. As shown in Supplementary Fig. S1A, ART inhibited Y-79 cells viability in a dose-dependent manner, the cell viability rates were as follows: 0.57 ± 0.09 (ART 10 μg/ml), 0.24 ± 0.03 (ART 20 μg/ml), 0.19 ± 0.04 (ART 40 μg/ml), 0.17 ± 0.04 (ART 80 μg/ml) (relative to the control: 1) (P<0.05). Subsequently, Y-79 cells were exposed to 20 µg/ml of ART for 24, 36 or 48h, the CCK-8 data showed that ART also exerted a time-dependent growth inhibition on Y-79 cells, the cell viability rates were as follows: 0.59 ± 0.01 (24h), 0.24 ± 0.03 (36h), 0.17 ± 0.02 (48h) (relative to the control: 1) (P<0.05) (Supplementary Fig. S1B).

**ART has limited inhibited effect in normal retina cells.**

To determine whether ART is harmful to the normal retina cells in vitro, cell viability of human retinal pigment epithelium cell line A-RPE 19 and primary rat retina neurons were measured after ART treatment (20 µg/ml) for 24, 36 or 48h. The results showed that ART slightly suppresses the cells growth, the cell viability rates were as follows: A-RPE 19: 0.93 ± 0.02 (24h), 0.86 ± 0.03 (36 h), 0.85 ± 0.04 (48h) (relative to the control: 1) (P<0.05) (Supplementary Fig. S2A); retina neurons: 0.92 ± 0.01(24h), 0.89 ± 0.04(36 h), 0.83 ± 0.02 (48h) (relative to the control: 1) (P<0.05) (Supplementary Fig. S2B).

**The inhibition of WERI-Rb1 cells proliferation by ART is mainly via inducing cell apoptosis.**

To confirm which pattern of cells inhibition is underlying after ART treatment, cell cycle, cell apoptosis and necrosis were analysis by flow cytometry. After WERI-Rb1 cells were treated with ART (40 μg/ml) or a vehicle control for 36h, the data showed that ART treatment significantly promoted cell apoptosis, the percentages of apoptotic WERI-Rb1 cells were 39.6 ± 9.2% compared to the control 9.7 ± 1.5%. Whereas, only a small mount of WERI-Rb1 cells were arrested at the S phase (Control: 21.96 ± 0.61%; ART: 29.48 ± 0.94%), only small quantity of necrosis of WERI-Rb1 cells were found, the necrosis rates were as follows: 5.49 ± 0.86% (control) and 9.1 ± 0.21% (ART) (Supplementary Fig. S3). Together, these results suggested that the anti-RB activity of ART mainly via inducing cell apoptosis.

**ART-induced apoptosis of Y-79 cells by upregulating KLF6 expression.**

In order to detect whether ART have the same effect on regulating KLF6 in Y-79 cell line, Y-79 cells were treated with various concentrations of ART (10, 20, 40 or 80 μg/ml) or a vehicle control for 24 h. Supplementary Fig. S4A showed that mRNA expression level of KLF6 was markedly upregulated after ART treatment in a dose-dependent manner by using real-time PCR (by 2.76-, 4.08-, 4.86-, and 5.36-fold compared with the control, respectively). Similarly, ART also promoted the KLF6 protein expression in a dose-dependent manner (by 1.91-, 2.65-, 2.95-, and 3.43-fold compared with the control, respectively) (Supplementary Fig. S4B-C). KLF6 protein expression also markedly decreased after transfected with siKLF6 (control: 1; ART: 3.71 ± 0.52; ART + siKLF6: 2.06 ± 0.13; P<0.05) (Supplementary Fig. S4D-E). Furthermore, silencing of KLF6 could significantly increase cell viability (control: 1; ART: 0.32 ± 0.02; ART + siKLF6: 0.61 ± 0.04; P<0.05) and attenuate the cell apoptosis rate (control: 6.00± 1.05%; ART: 56.50 ± 14.71%; ART + siKLF6: 23.60 ± 2.86; P<0.05) following ART treatment in Y-79 cells (Supplementary Fig. S4F-G). Caspases-9 and-3 protein expression also detected after silence of KLF6 followed by ART treatment. The results showed that the cleaved caspase-3 and cleaved caspase-9 expression are increased after TMP treatment, however, silencing of KLF6 significantly inhibits this effect (Supplementary Fig. S4H-I). Taken together, these results suggested that KLF6 plays a crucial role in ART-induced apoptosis of Y-79 cells.

**Overexpression of KLF6 promotes WERI-Rb1 cells apoptosis.**

To confirm KLF6 overexpression plays an important role in WERI-Rb1 cells apoptosis, cells were transfected with FLAG-KLF6 or vector pCI-neo plasmids. The expression of KLF6 was assessed by western blot analysis. Relative quantification data revealed that KLF6 expression was increased (Vector: 1; KLF6: 1.44 ± 0.15; P<0.05) (Supplementary Fig. S5A-B). Then, the cell viability was measured and the result exhibited the inhibition after transfected with flag-KLF6 (relative cell viability value: Vector: 1; KLF6: 0.76 ± 0.3; P<0.05) (Supplementary Fig. S5C). What’s more, the flow cytometry results also showed that the overexpression of KLF6 increases the cell apoptosis rates compared with those of the null control group cells (the percentages of apoptotic cells: Vector: 13.08 ± 3.8%; KLF6: 28.7 ± 5.08%; P<0.05) (Supplementary Fig. S5D-E). Taken together, these results suggested that overexpression of KLF6 promotes WERI-Rb1 cells apoptosis.

**Figures**


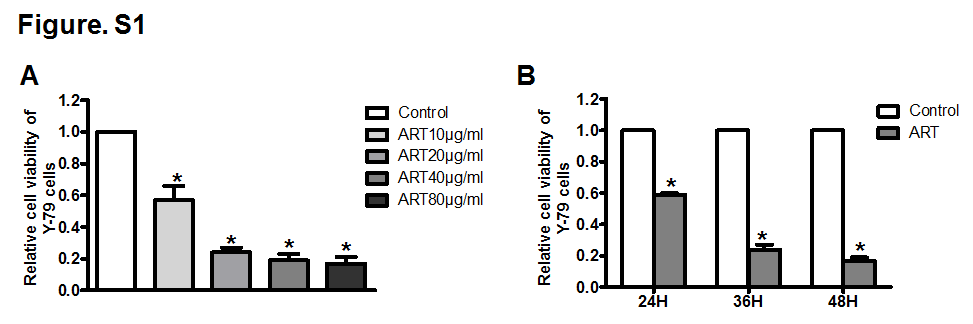


**Supplementary Fig. S1**


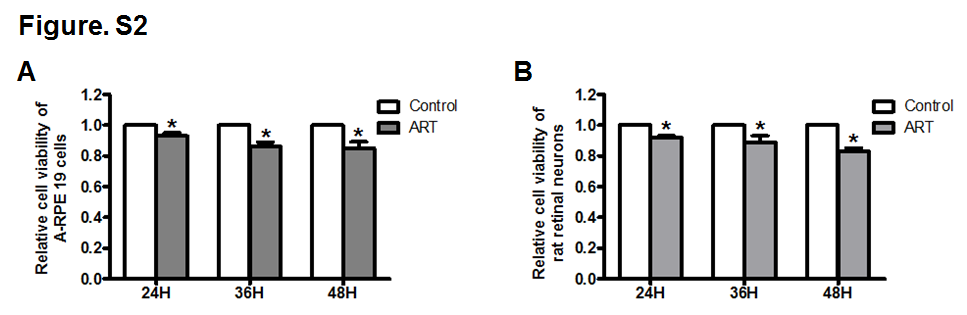


**Supplementary Fig. S2**


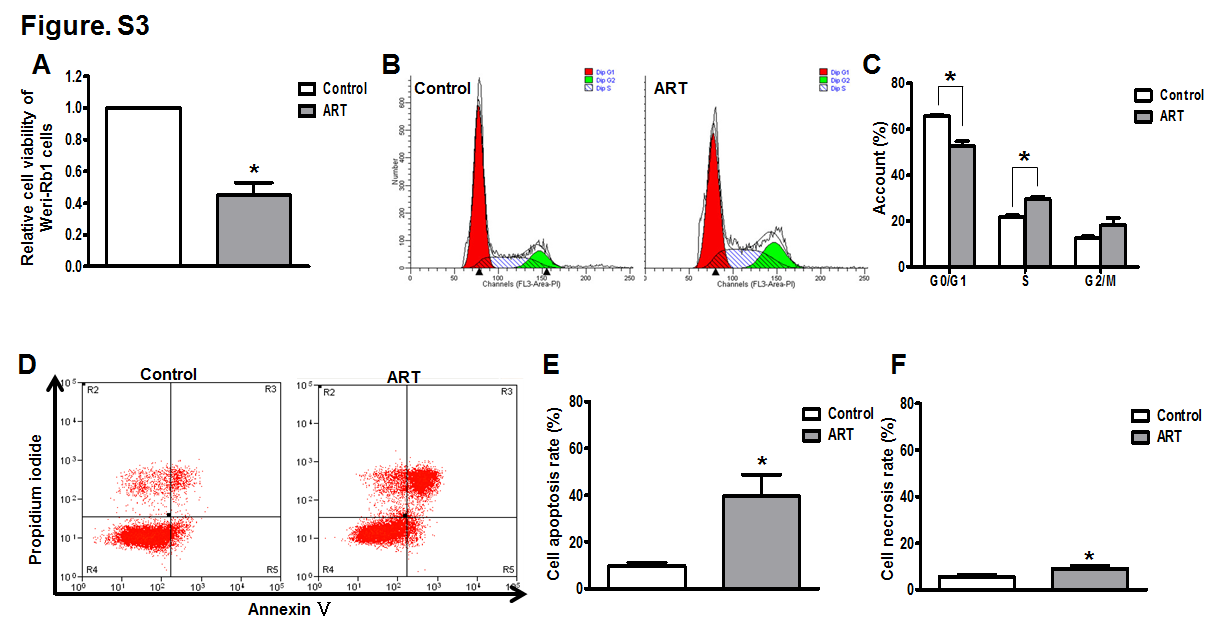


**Supplementary Fig. S3**


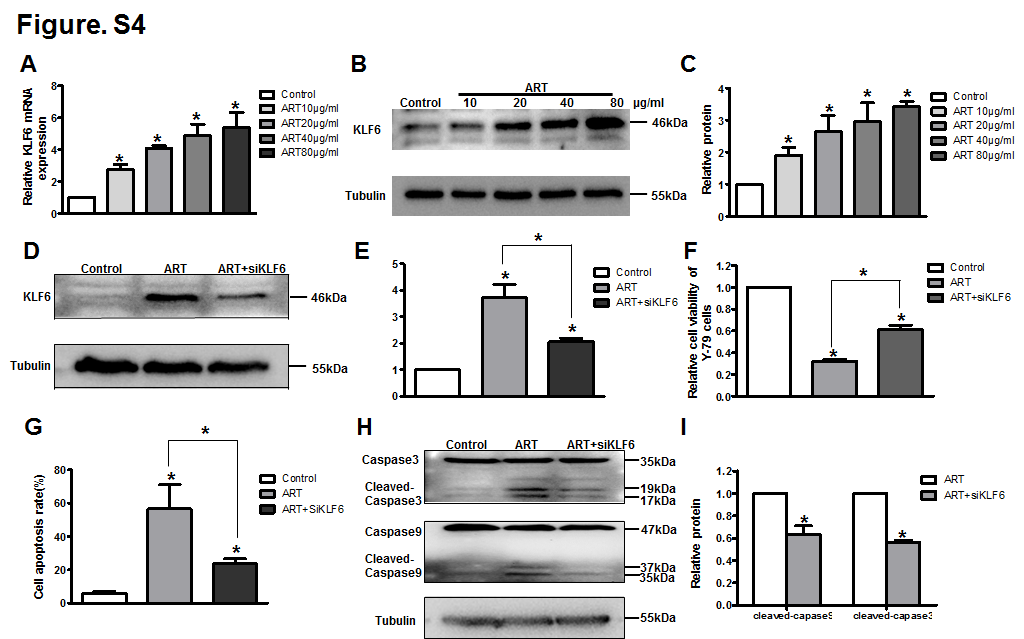


**Supplementary Fig. S4**


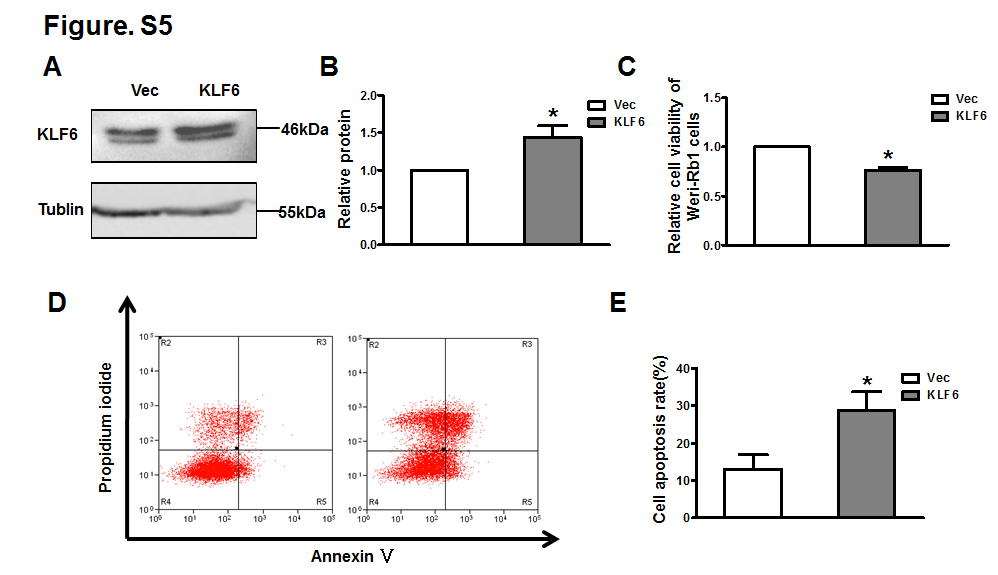


**Supplementary Fig. S5**


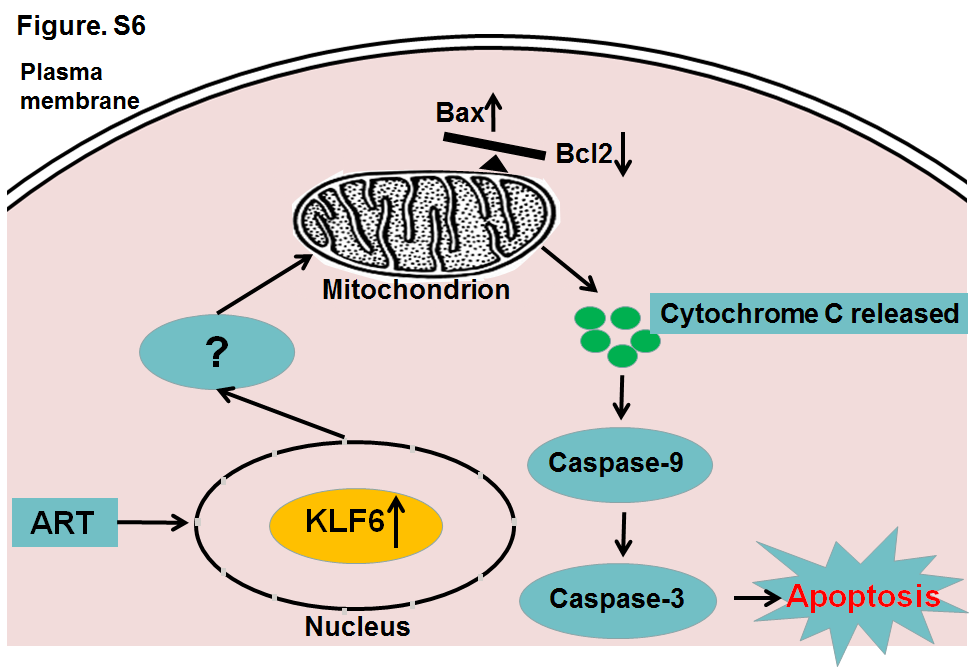


**Supplementary Fig. S6**
