# Supplementary material for: Artesunate induces mitochondria-mediated apoptosis of human retinoblastoma cells by upregulating Kruppel-like factor 6
Source: Cell Death Dis. 2019 Nov 13;10(11):862. doi: 10.1038/s41419-019-2084-1 (PMC6853908; doi:10.1038/s41419-019-2084-1)
Supplement: Supplementary file 2 — Supplementary Figure Legends [file 41419_2019_2084_MOESM2_ESM.docx]

**Supplementary Fig. S1** ART inhibits Y-79 cells proliferation. (A) Cultured Y-79 cells were exposed to various concentrations (10, 20, 40 or 80 μg/ml) of ART for 36 h, CCK-8 assay was performed to evaluate the cell viability. ART could significantly inhibit cells viability a dose-dependent manner. (B) Y-79 cells were exposed to 20 µg/ml of ART for 24, 36 or 48h. CCK-8 data showed that ART also exerted a time-dependent growth inhibition in Y-79 cells. All results are presented as the mean ± SD (n=3, *P<0.05).

**Supplementary Fig. S2** ART has limited inhibited effect in normal retina cells. Cultured human retinal pigment epithelium cell line A-RPE 19 cells (A) and primary rat retina neurons (B) were exposed to 20 µg/ml of ART for 24, 36 or 48h. CCK-8 data showed that ART slightly suppresses the cells growth. All results are presented as the mean ± SD (n=3, *P<0.05).

**Supplementary Fig. S3** ART inhibits WERI-Rb1 cells proliferation mainly via inducing cells apoptosis. The WERI-Rb1 cells were treated with ART (40 μg/ml) or a vehicle control for 36h. (A) Cell viability was analyzed by the CCK-8 assay, ART could significantly suppress cells growth. (B) Cell cycle profiles were analyzed by flow cytometry, (C) data demonstrated that a small amount of WERI-Rb1 cells are arrested at the S phase. (D) Apoptotic and necrotic WERI-Rb1 cells were calculated by flow cytometry after annexin V and propidium iodide staining. (E) ART treatment greatly promoted cell apoptosis. (F) ART treatment also induced the minimal necrosis of WERI-Rb1 cells. All results are presented as the mean ± SD (n=3, *P<0.05).

**Supplementary Fig. S4** ART-induced apoptosis of Y-79 cells by up-regulating KLF6. (A) The mRNA expression level of KLF6 in Y-79 cells was markedly upregulated after ART treatment in a dose-dependent manner by using real-time PCR. (B-C) KLF6 protein expression in Y-79 cells were detected and quantified, the results showed KLF6 protein was increased after ART treatment in a dose-dependent manner. (D-E) KLF6 protein was downregulated after transfected with KLF6 siRNA. KLF6 silencing attenuates the effect of ART on inhibiting cell viability (F) and promoting cell apoptosis rate (G) of Y-79 cells. (H-I) Silencing of KLF6 significantly inhibited cleavage of caspases-9 and -3 detected by western blot and quantified. All results are presented as the mean ± SD (n=3, *P<0.05).

**Supplementary Fig. S5** Overexpression of KLF6 inhibits WERI-Rb1 cell growth and promotes cell apoptosis. Cultured WERI-Rb1 cells were transfected with plasmid FLAG-KLF6. (A) The protein expression levels of KLF6 was examined by western blot analysis. (B) Relative quantification data revealed that KLF6 expression was increased. (C) Effect of KLF6 overexpression on cell proliferation was determined by the CCK-8 assay. Cell viability was inhibited after transfection. (D-E) Flow cytometry revealed that overexpression of KLF6 increased the rate of cell apoptosis. All results are presented as the mean ± SD (n=3, *P<0.05, vs. control).

**Supplementary Fig. S6** The mechanism diagram underlying this study. ART treatment upregulates KLF6 expression, which causes mitochondria dysfunction, increases the Bax/Bcl-2 ratio, promotes the release of cyt-c, and stimulate the cleavage of caspase-9 and caspase-3, resulting in cell apoptosis.
