# Supplementary material for: Artesunate induces mitochondria-mediated apoptosis of human retinoblastoma cells by upregulating Kruppel-like factor 6
Source: Cell Death Dis. 2019 Nov 13;10(11):862. doi: 10.1038/s41419-019-2084-1 (PMC6853908; doi:10.1038/s41419-019-2084-1)
Supplement: Supplementary file 3 — author contribution [file 41419_2019_2084_MOESM3_ESM.pdf]

DECLARATION OF CONTRIBUTIONS TO ARTICLE

ADMC

Manuscript Number:	Journal Name:	
CDDIS-19-1261R	*Cell Death & Differentiation*	(the 'Journal')

Proposed Title of the Contribution:

Artesunate induces mitochondria-mediated apoptosis of human retinoblastoma cells by upregulating Kruppel-like factor 6 (the 'Contribution)

Author(s):

Ying Yang, Nandan Wu, Yihui Wu, Haoting Chen, Jin Qiu, Xiaobing Qian, Jieting Zeng, Kin Chiu, Qianying Gao, Jing Zhuang (the 'Authors')

For all *CDD* articles, each person named as an author in the published version must be able to show he or she has contributed substantially to the article.

Authorship credit should be based on 1) substantial contributions to conception and design, acquisition of data, or analysis and interpretation of data; 2) drafting the article or revising it critically for important intellectual content; and 3) final approval of the version to be published. Authors should meet conditions 1, 2 and 3.

Any person who cannot be shown to have made a substantial contribution to the article cannot be listed as an author in the final version. The name of any person who is deemed to have made a minor contribution can, however, appear in the Acknowledgments section of the article.

Please complete the table below to indicate the contributions of all named authors to the manuscript.

Author Full Name:	Specification of Contribution to the Manuscript:
Ying Yang	YY designed the study, performed experiments, collected and analyzed data, and wrote the manuscript.
Nandan Wu	NW designed the study, performed experiments, collected and analyzed data, and wrote the manuscript.
Yihui Wu	YW performed experiments and collected data.
Haoting Chen	HC performed experiments and analyzed data.
Jin Qiu	JQ performed experiments and helped analyze data.
Xiaobing Qian	XQ helped collected data and assembled the figure.
Jieting Zeng	JZ helped collected and analyzed data.
Kin Chiu	KC helped designed the study of animals and revised the manuscript.
Qianying Gao	QG designed the study, analyzed data and revised the manuscript.
Jing Zhuang	JZ designed the study, analyzed data and revised the manuscript.

Please complete the table below to indicate the contributions of all named authors to the figures.

Figure 1:

In Figure 1, YY and NW performed CCK-8, flow cytometry experiments,analyzed the data and assembled the figure.

Figure 2:

In Figure 2, YY and JQ collected and analyzed data of genes microarray (Fig. 2 A, B). NW performed the western blot experiments and JZ (Jieting zeng) helped analyze the data (Fig. 2 C, D, E). XQ assembled the figure.

Figure 3:

In Figure 3, YY performed the immunehistochemistry and CCK-8 experiments(Fig. 3A, G). YW performed the cell culture and siRNA transfection. NW and HC performed RT-PCR, western blot and flow cytometry experiments (Fig. 3B-H). NW analyzed the data and assembled the figure.

Figure 4:

In Figure 4, YW performed the cell culture, siRNA transfection and drug treatment, YY performed western blot experiments, analyzed the data and assembled the figure.

Figure 5:

In Figure 5, YY and NW estabished the orthotopic xenotransplantation model of RB, collected and analyzed the tumor size and weight, performed western blot experiments and analyzed the data(Fig. 5A-E, G). YW performed the TUNEL assay(Fig. 5F).

Figure 6:

In Figure 6, YY and NW established the subcutaneous xenotransplantation model of RB, collected and analyzed the tumor volume and weight; KC provided the expertise on lentiviral and animals study(Fig. 6A-D). JQ performed western blot experiments(Fig. 6E). YW performed the TUNEL assay(Fig. 6F).

Signed for and on behalf of the Author(s):	Print Name:	Date:
Jingzhuang	Jing Zhuang	2019-10-10

There is not enough room in the author contribution form, so we list it here as follows:

Figure 7

In Figure 7, YY and NW performed the color fundus photography, FFA, ERG (electroretinogram) and OCT examination (Fig.7A-D); YW and HC helped collected and analyzed the data, assembled the figure (Fig.7B, C).

Supplemental Figure S1

In Supplemental Figure S1, YY performed CCK-8 assay, analyzed the data and assembled the figure.

Supplemental Figure S2

In Supplemental Figure S2, YY performed CCK-8 assay, analyzed the data and assembled the figure.

Supplemental Figure S3

In Supplemental Figure S3, NW performed the flow cytometry experiments, analyzed the data and assembled the figure.

Supplemental Figure S4

In Supplemental Figure S4, YW performed the cell culture, siRNA transfection; NW performed the western blot experiments, JZ (Jieting zeng) helped analyze the data and assembled the figure.

Supplemental Figure S5

In Supplemental Figure S5, YW performed the cell culture, plasmid transfection; NW performed the western blot and flow cytometry

experiments, analyzed the data and assembled the figure.

Supplemental Figure S6

In Supplemental Figure S6, QG and JZ (Jing zhuang) elaborated the mechanisms of ART-induced apoptosis on RB cells in the form of schematic diagram.
